# Unlocking the Therapeutic Potential of Ulotaront as a Trace Amine-Associated Receptor 1 Agonist for Neuropsychiatric Disorders

**DOI:** 10.3390/biomedicines11071977

**Published:** 2023-07-13

**Authors:** Savelii R. Kuvarzin, Ilya Sukhanov, Kirill Onokhin, Konstantin Zakharov, Raul R. Gainetdinov

**Affiliations:** 1 Institute of Translational Biomedicine, Saint Petersburg State University, 199034 Saint Petersburg, Russia; 2 Valdman Institute of Pharmacology, Pavlov Medical University, 197022 Saint Petersburg, Russia; 3 Accellena Research and Development Inc., 199106 Saint Petersburg, Russia; 4 Saint Petersburg University Hospital, Saint Petersburg State University, 199034 Saint Petersburg, Russia

**Keywords:** SEP-363856, antipsychotic agents, dopamine, schizophrenia, ulotaront, trace amine-associated receptor 1, TAAR, neuropsychiatric disorders

## Abstract

All antipsychotics currently used in clinic block D2 dopamine receptors. Trace amine-associated receptor 1 is emerging as a new therapeutic target for schizophrenia and several other neuropsychiatric disorders. SEP-363856 (International Nonproprietary Name: Ulotaront) is an investigational antipsychotic drug with a novel mechanism of action that does not involve antagonism of dopamine D2 receptors. Ulotaront is an agonist of trace amine-associated receptor 1 and serotonin 5-HT1A receptors, but can modulate dopamine neurotransmission indirectly. In 2019, the United States Food and Drug Administration granted Breakthrough Therapy Designation for ulotaront for the treatment of schizophrenia. Phase 2 clinical studies indicated that ulotaront can reduce both positive and negative symptoms of schizophrenia without causing the extrapyramidal or metabolic side effects that are inherent to most currently used antipsychotics. At present, it is in phase 3 clinical development for the treatment of schizophrenia and is expected to be introduced into clinical practice in 2023–2024. Clinical studies evaluating the potential efficacy of ulotaront in Parkinson’s disease psychosis, generalized anxiety disorder, and major depressive disorder have also been started. The aim of this scoping review is to summarize all currently available preclinical and clinical evidence on the utility of ulotaront in the treatment of schizophrenia. Here, we show the main characteristics and distinctive features of this drug. Perspectives and limitations on the potential use of ulotaront in the pharmacotherapy of several other neuropsychiatric disorders are also discussed.

## 1. Introduction

Schizophrenia is a chronic psychiatric disorder that affects about 1% of the population worldwide and is characterized by continuous or relapsing episodes of psychosis. The major manifestations of schizophrenia include positive symptoms (hallucinations, various delusions, paranoia, and disorganized thinking), negative symptoms (social withdrawal, decreased emotional expression, anhedonia, alogia, and avolition), and cognitive deficits (deficient memory and impaired attention and executive functions) [1]. It is believed that the major contributors to the neuropathophysiology of this disorder are excessive dopaminergic activity and deficits in cortical glutamatergic neurotransmission, but it appears to be much more complex, involving structural and molecular changes throughout brain circuits involving alterations in other neurotransmitters, monoamines, and their derivatives [2,3,4,5,6,7,8,9,10,11].

Following the serendipitous discovery of the antipsychotic action of chlorpromazine in the 1950s, many antipsychotics have been introduced into clinical practice. However, all of them essentially share the same mechanism(s) of action, involving antagonism or partial agonism of the dopamine D2 receptor (D2R). Traditionally, antipsychotics are divided into two generations. The drugs of the first generation (‘typical’) are thought to work mainly by the blockade of D2R and often induce extrapyramidal symptoms in patients. The drugs of the second generation (‘atypical’) share the ability to block the serotonin receptor 2A subtype (5-HT2A), in addition to D2R, and their safety profiles are sometimes better. Additionally, some clinicians distinguish atypical antipsychotics that are partial D2R agonists, such as aripiprazole, brexpiprazole, cariprazine, and lumateperone, as a third generation of these drugs. However, the efficacies of typical and atypical antipsychotics do not differ strongly and mostly affect positive symptoms, with negative symptoms and cognitive deficits remaining essentially untreatable [12,13]. Additional limitations in the clinical use of both first- and second-generation antipsychotics include adverse effects, particularly extrapyramidal motor effects and metabolic dysregulations, which often lead to nonadherence to treatment [14]. Pimavanserin, a 5-HT2A serotonin receptor blocker, is the only antipsychotic without D2R antagonistic properties, but it failed in schizophrenia trials and is currently approved only for Parkinson’s disease psychosis [15]. Therefore, there is an urgent need for new medications with novel mechanisms of action that do not involve D2R and/or 5-HT2A serotonin receptor antagonism for the treatment of schizophrenia.

One of the approaches used to develop such drugs is the target-agnostic in vivo phenotypic drug discovery protocol [16]. Sunovion Pharmaceuticals partnered with PsychoGenics Inc. to use its proprietary, high-throughput SmartCube^®^ platform that combines in vivo behavioral testing with artificial intelligence to phenotypically discover potential anti-psychotics. This approach resulted in the discovery of the antipsychotic-like profile of the Sunovion Pharmaceuticals compound ulotaront (INN; developmental codes: SEP-363856, SEP-856). By testing the radioligand binding profile, ulotaront was initially characterized as a serotonin 5-HT1A agonist; however, follow-up functional studies revealed the potent agonistic action of the compound at Trace Amine-Associated Receptor 1 (TAAR1), its potency being one order of magnitude higher than that at the serotonin 5-HT1A receptor [17].

Identified in 2001, Trace Amine-Associated Receptors (TAARs—six functional receptors found in humans: TAAR1, TAAR2, TAAR5, TAAR6, TAAR8, and TAAR9) comprise a family of G protein-coupled receptors that are clustered in a chromosomal region associated with schizophrenia [18,19,20]. While TAAR2-TAAR9 receptors were initially considered as a new class of olfactory receptors sensing innate odors mediated by volatile amines [21], recent observations indicate that at least some of these receptors are expressed also in the limbic brain areas and involved in emotional regulation and adult neurogenesis [22,23,24]. The best-studied receptor is TAAR1, which is expressed in part within the dopaminergic neuronal circuitry and can be activated by a variety of monoaminergic compounds, including trace amines, amphetamines, and monoamine metabolites. TAAR1 is emerging as a promising new target for psychiatric disorders [25,26,27]. Pharmacological or genetic targeting of TAAR1 revealed that stimulation of TAAR1 suppressed dopamine-dependent behaviors, while TAAR1 deficiency potentiated them [26,28,29]. This modulation likely involves the regulation of striatal presynaptic and postsynaptic D2R function via D2R-TAAR1 heterodimerization and modulation of the beta-arrestin2-dependent Akt/GSK3beta signaling cascade [30,31,32]. Thus, instead of acting on D2R directly, ulotaront may affect its functions indirectly through activation of the TAAR1 heterodimer, thereby providing a novel opportunity to bias D2R signaling [33].

TAAR1 seems to affect the function of the other critical brain neurotransmitters. A distinct pattern of expression of TAAR1 in the pyramidal neurons of layer V of the prefrontal cortex (PFC) was detected, and altered subunit composition and deficient functionality of the glutamate NMDA receptors in the PFC and striatum were found in mice lacking TAAR1 [34,35]. These studies indicate that TAAR1 plays an important role in the modulation of dopamine-related processes in the striatum and NMDA receptor-mediated glutamate transmission in the PFC. TAAR1 is also found in the serotonergic dorsal raphe nucleus (DRN) and regulates serotonin transmission [28]. By modulating dopamine, glutamate, and serotonin transmission, selective TAAR1 agonists have shown potential antipsychotic, antidepressant, and pro-cognitive effects in several experimental animal models [36,37]. Thus, these data suggest that the development of TAAR1-based drugs could provide a novel therapeutic approach for the treatment of neuropsychiatric disorders related to aberrant frontostriatal circuitry. Based on the preclinical studies, it might be expected that TAAR1 could be a potential drug target for several neuropsychiatric disorders, including schizophrenia, depression, bipolar disorder, generalized anxiety disorder, addiction, ADHD, Alzheimer’s disorder, etc. [29,37,38,39,40,41,42,43,44,45,46,47,48,49,50,51,52].

To date, two TAAR1 agonists, ralmitaront (F. Hoffmann La-Roche, phase 2, Clinical-Trials.gov identifier: NCT03669640) and ulotaront (SEP-363856, 1-[(7S)-5,7-dihydro-4H-thieno [2,3-c]pyran-7-yl]-N-methylmethanamine, Sunovion Pharmaceuticals, phase 3, described below), are undergoing clinical trials in schizophrenia treatment. In 2019, based on the results of a phase 2 clinical trial, ulotaront was awarded FDA Breakthrough Therapy Designation for schizophrenia treatment due to its demonstrated efficacy and greatly reduced side effects compared to current treatments [53,54].

Here, we present a summary of the accumulating data from preclinical and clinical investigations of ulotaront, highlighting its potential for the treatment of schizophrenia and other neuropsychiatric disorders, such as depression, generalized anxiety disorder, Parkinson’s disease psychosis, and others.

## 2. Methods

### 2.1. Search Strategy

The PubMed, ISI Web of Science, and Google Scholar electronic databases were searched from inception to June 2023. The specific search terms were “SEP-363856”, “SEP-856”, and “ulotaront”. In addition, all clinical trials containing the drug name “SEP-363856” were obtained from the ClinicalTrials.gov registry. The data and experimental results from the publications we found were extracted independently by two of the authors.

### 2.2. Inclusion and Exclusion Criteria

All peer-reviewed original research articles and conference abstracts were included for further analysis of the results. Reviews found by searching for the listed terms were excluded.

### 2.3. Synthesis of Results

The main findings of the preclinical and clinical studies were analyzed and systematized in the relevant sections of this review.

## 3. Results

Based on our search, we found twenty preclinical and clinical original research articles, as well as twenty-six clinical trials in phases 1–3 in the ClinicalTrials.gov registry. Here, we provide a review of all currently available in vitro and in vivo preclinical data and pharmacokinetic properties and a description of all the performed and ongoing clinical studies indicating the long-term efficacy of ulotaront against both positive and negative symptoms of schizophrenia with minimal extrapyramidal and metabolic side effects. The potential use of ulotaront in the treatment of other neuropsychiatric disorders is also discussed.

## 4. Discussion

Below, we have systematized the experimental data we found by dividing them into several subsections. The results of the preclinical and clinical studies are discussed separately, and the clinical trials that have been initiated and whose results have not yet been published are also described.

## 5. Summary of Evidence

### 5.1. Preclinical Pharmacology

#### 5.1.1. In Vitro Pharmacological Studies in Heterologous Cellular Cultures

In an initial radioligand binding screening assay, ulotaront (at 10 µM) showed >50% inhibition of specific binding at the 5-HT1A, 5-HT1B, 5-HT1D, 5-HT2A, 5-HT2B, 5-HT2, 5-HT7, α2A, α2B, and D2R receptors. Ki values varied from 0.031 to 21 µM. In follow-up functional assays, it was identified as an agonist of the human TAAR1 receptor, with an EC50 of 0.14 ± 0.062 μM and an Emax of 101.3 ± 1.3% (means ± SEMs), and the 5-HT1A receptor, with an EC50 of 2.3 ± 1.40 μM and an Emax of 74.7 ± 19.60%. Weak effects on all other targets (the 5-HT1B, 5-HT1D, 5-HT2A, 5-HT2B, 5-HT2, 5-HT7, α2A, α2B, and D2 receptors) were observed only at high micromolar concentrations. In D2R receptor functional assays, ulotaront exhibited weak partial agonist activity, with EC50 values of 10.44 ± 4 µM (cAMP, Emax = 23.9% ± 7.6%) and 8 µM (β-arrestin recruitment, Emax = 27.1%). At 100 µM, 34% ± 1.16% inhibition was seen in the cAMP assay, and no antagonism was seen at concentrations up to 100 μM in the β-arrestin recruitment assay. Low potency partial agonist activities were also observed at the 5-HT1B (EC50 = 15.6 ± 11.6 μM, Emax = 22.4% ± 10.9%), 5-HT1D (EC50 = 0.262 ± 0.09 μM, Emax = 57.1% ± 6.0%), and 5-HT7 receptors (EC50 = 6.7 ± 1.32 μM, Emax = 41.0% ± 9.5%). In a functional assay of 5-HT2B activity, ulotaront showed no agonism up to a concentration of 100 µM. Little to no activity was detected at the 5-HT2A receptor, with 29.3% agonism seen only at the highest tested concentration of 10 μM. Ulotaront did not show activity in any of the studied enzymes up to a concentration of 100 µM [17]. Intriguingly, a more recent study involving an alternative in vitro cellular method of detecting TAAR1 activity revealed a more potent (EC50 of 38 ± 11 nM, Emax = 109% ± 3%) agonistic action of ulotaront, thereby revealing a higher selectivity level with respect to 5-HT1A activity [55]. A similar difference in activity at TAAR1 vs. 5-HT1A was shown in an independent study [56]. It has been shown that ulotaront is a potent full agonist of TAAR1, acting specifically via Gαs recruitment (pEC50 = 6.08 ± 0.22, Emax = 96.41% ± 15.26). Ulotaront showed slightly less potency than β-PEA (pEC50 = 6.49 ± 0.23) against TAAR1, but greater potency than p-Tyramine (pEC50 = 5.65 ± 0.06). At the same time, ulotaront demonstrated partial agonist activity at the 5-HT1A receptor, with a potency that was several orders of magnitude lower. Similarly to the endogenous agonist, at extremely high micromolar concentrations, ulotaront could induce 5-HT1A-dependent recruitment of Gαq, Gαi, and, to a lesser extent, Gαs (Emax = 36–47%) [56]. It has also been shown that ulotaront can affect 5-HT1A-mediated GIRK activity. The ability of TAAR1 agonists to activate GIRK via TAAR1 was demonstrated previously [28,57]. Ulotaront induced Gβγ-mediated GIRK activation (Emax = 55.03% ± 14.09), indicating that the compound at high micromolar concentrations can induce GIRK channel-mediated currents through the 5-HT1A receptor, presumably due to Gαi/o coupling [56]. However, whether these high concentrations (>100 µM) of ulotaront required for activity at 5-HT1A have any physiological relevance remains unclear. Furthermore, the activity of ulotaront at D2R was estimated. In agreement with previous results [17], ulotaront demonstrated only low potency and efficacy activity at D2R with regard to Gαi recruitment and GIRK activation at extremely high (up to 1 mM) concentrations.

#### 5.1.2. Studies on Neuronal Tissues

To evaluate the mechanisms responsible for the action of ulotaront at the neuronal level, whole-cell patch-clamp recordings in isolated mouse brain slices of the dorsal raphe nucleus (DRN), where cell bodies of serotonin neurons are located, and the ventral tegmental area (VTA) containing cell bodies of mesolimbic dopaminergic neurons were performed. Ulotaront induced inhibitory responses in DRN neurons, and this effect was attenuated by the 5-HT1A antagonist WAY-100635 but not by the TAAR1 antagonist EPPTB. VTA neuron activity was also reduced by ulotaront: inhibitory effects in VTA were attenuated by the TAAR1 antagonist EPPTB but not the 5-HT1A antagonist WAY-100635. It seems that the inhibitory effects of ulotaront on the activity of DRN neurons were mediated via the activation of serotonin 5-HT1A receptors but that in the VTA neurons they were at least partially dependent on TAAR1 activation [17]. Analysis of extracellular single-unit activities of the DRN neurons of anesthetized rats corroborated the findings gained via whole-cell patch-clamp recordings in isolated mouse brain slices. At a dose of 5 mg/kg (i/v), ulotaront completely suppressed neuron firing, and this inhibition was fully reversed by the serotonin 5-HT1A antagonist WAY-100635, indicating that the inhibitory effects of ulotaront on DRN neurons are mediated exclusively through serotonin 5-HT1A receptors [17]. To directly evaluate the action of ulotaront on serotonin 5-HT1A receptors in brain tissue in vitro, autoradiography in rat brain slices was performed. Serotonin 5-HT1A agonist 8-OH-DPAT radioligand binding in the absence and presence of ulotaront was quantified. Ulotaront displaced 8-OH-DPAT in a concentration-dependent manner, and the highest receptor binding was observed in the septum and throughout the cortex [17].

To assess ulotaront occupancy at D2R, in vivo autoradiography experiments with the radioligand D2R antagonist raclopride in Sprague Dawley rats were carried out. Ulotaront did not produce significant occupancy at D2R in the brain at plasma concentrations 200-fold greater than those that were behaviorally effective, and no significant interaction of the drug with D2R was shown [17]. Furthermore, PET imaging of fallypride radiotracers in anesthetized baboons was conducted to determine D2R occupancy in primates. Ulotaront, even at very high concentrations, showed very low D2R occupancy levels (less than 10%) in brain regions. The lack of direct D2R interaction appears to extend to primates as well [17].

Taken together, these data indicate that ulotaront can act predominantly via the activation of TAAR1 and serotonin 5-HT1A receptors without a significant effect on D2R and serotonin 5-HT2A receptors.

### 5.2. In Vivo Behavioral Studies

#### 5.2.1. Antipsychotic Action: Positive Symptoms

Some of the most widely accepted pathogenetic hypotheses of schizophrenia are increased dopaminergic and decreased glutamatergic transmission [58,59]. Drugs acting through an increase in dopamine or a decrease in glutamate signaling are usually applied for modeling schizophrenia endophenotypes in rodents and for testing potential drugs in these models. Positive symptoms are usually modeled in tests involving the locomotor hyperactivity of mice and rats following administration of the dopaminergic stimulant amphetamine or the glutamate NMDA receptor antagonist phencyclidine (PCP) [60].

Ulotaront showed good efficacy in a PCP-induced hyperactivity psychosis model, which was used for modeling positive symptoms. All tested doses (0.3, 1, and 3 mg/kg, p/o) decreased the hyperlocomotion of mice in a dose-dependent manner [17]. The attenuation of PCP-induced hyperlocomotion was also observed in rats, with a minimal effective dose of 1 mg/kg, p/o. The serotonin 5-HT1A receptor antagonist WAY-100635 partially decreased the ability of ulotaront to attenuate PCP-induced hyperactivity in mice [17]. The potential role of the 5-HT1A mechanism of ulotaront in its action on PCP-induced hyperactivity was further supported recently in mice lacking TAAR1: pretreatment with the drug (10 mg/kg, p/o) diminished MK-801-induced hyperactivity independently of TAAR1 [56].

Intriguingly, ulotaront failed to mitigate locomotor hyperactivity induced by dopaminergic drugs, indicating the complex action of the agent on dopamine neurotransmission and emphasizing the non-D2R mechanism(s) involved. Thus, pretreatment with the agent (dose range: 1–10 mg/kg, p/o) did not reverse d-amphetamine-induced hyperlocomotion [56,61]. Similar data were obtained when the effect of ulotaront was evaluated in the dopamine agonist apomorphine-induced climbing test in mice [62]. At the same time, treatment with ulotaront potentiated effects of the antipsychotic drug olanzapine in this test as well as in the NMDA antagonist MK-801-induced hyperactivity test in mice [62].

#### 5.2.2. Antipsychotic Action: Negative Symptoms and Cognitive Deficits

One of the commonly used negative symptom models is decreased social interaction in rodents induced by chronic PCP administration [63,64]. Ulotaront was effective against sub-chronic PCP-induced deficits in the social interaction test in rats. All tested doses of ulotaront increased social interaction time, the magnitude of the effect tending to decrease as the dose increased from 1 to 10 mg/kg [61]. Ulotaront at 10 mg/kg also ameliorated cognitive impairments caused by sub-chronic treatment with PCP in the novel object recognition test in rats [61]. Furthermore, ulotaront slightly mitigated MK-801-induced deficits in the Morris water maze test and potentiated the ameliorative effect of olanzapine in this cognitive assay [62]. Deficits in sensorimotor gating are present in patients suffering from schizophrenia, and these deficits can be modeled in rodents. Studies of drug effects in the pre-pulse inhibition (PPI) test of the acoustic startle response in rodents showed good utility in the identification of potential antipsychotic medications [65]. Ulotaront at doses of 0.3–30 mg/kg, p/o, dose-dependently increased PPI compared with the vehicle, with a minimal effective dose of 3 mg/kg [17]. These observations were supported further by an independent group [56]. The administration of ulotaront at a dose of 10 mg/kg, p/o, increased PPI and, most importantly, restored PPI disrupted by pretreatment with MK-801 in wild-type but not TAAR1-knockout mice [56]. These results indicate that ulotaront’s action on negative symptoms is directly related to its TAAR1 agonistic activity.

#### 5.2.3. Common Adverse Reactions of Antipsychotics

Extrapyramidal symptoms and weight gain are among the common adverse reactions induced by antipsychotic therapy. The catalepsy bar test was carried out to assess ulotaront’s potential to cause the development of extrapyramidal symptoms; haloperidol was used as a positive control. Ulotaront, at the highest studied dose of 100 mg/kg, p/o, produced no effect in mice, indicating the low potential of the drug to induce cataleptic effects at doses much higher than efficacious doses in psychosis models in mice [17]. However, ulotaront (10 mg/kg, p/o) reduced the basal locomotor activity of mice [56]. The last effect seems to be TAAR1-dependent because the agent did not affect locomotor activity in TAAR1-knockout mice [46]. Ulotaront’s effect on animal weight was also estimated in mice. It has been demonstrated that chronic treatment with the agent (dose range: 2–3 mg/kg, p/o) was not associated with weight gain [62]. Moreover, administration of ulotaront (3 mg/kg, p/o) prevented weight gain in animals chronically treated with olanzapine [62].

#### 5.2.4. Other Effects of Ulotaront

The use of second-generation antipsychotics as adjunctive agents in the therapy of depression is well known [66,67]. An analysis of ulotaront’s effects in the forced swim test (FST), a routine animal test performed to estimate the antidepressant-like activity of pharmacological agents, revealed that administration of the compound (dose range: 1–10 mg/kg, p/o) resulted in a reduction in immobility time in mice, indicating that ulotaront may have some antidepressant-like action [17]. Recently, these findings were further corroborated by another group [68]. In this study, ulotaront decreased immobility time in both the FST and its analog, the tail suspension test, as well as the potentiated effects of the antidepressant duloxetine [68]. Moreover, ulotaront (15 mg/kg, p/o, for 21 days) mitigated modeled anhedonia-like states induced by chronic mild unpredictable stress in the sucrose preference test, with no effect in non-stressed mice [68].

Ulotaront was tested in rats to determine its effect on sleep architecture. Ulotaront at doses of 1, 3, and 10 mg/kg, p/o, produced a dose-dependent decrease in rapid eye movement (REM) sleep, an increase in latency to REM sleep, and an increase in cumulative wake time. Ulotaront did not affect the cumulative non-REM time and latency to non-REM. Taken together, these results suggest that ulotaront can improve vigilance when administered during the inactive phase [17].

The development of any new psychotropic agents always raises questions about their addictive potential. In a recently published work, Synan and colleagues addressed this issue [69]. In this study, ulotaront was not able to maintain the self-administration (SA) of d-amphetamine, heroin, or cocaine, or serve as a substitute for cocaine in a drug discrimination paradigm. However, the compound can partly substitute for MDMA in drug discrimination tests, although the effect was demonstrated only for an extremely high (30 mg/kg, p/o) dose. Moreover, ulotaront (10 mg/kg, p/o) was found to be able to mitigate que- (but not prime-)induced reinstatement of cocaine SA. Intriguingly, ulotaront, as well as TAAR1 agonists in other studies ([70]; Dravolina et al., unpublished), inhibited food-reinforced behavior [69].

Importantly, ulotaront attenuated the ketamine-induced increase in the striatal dopamine synthesis capacity in mice without producing an effect in drug-naïve controls, indicating that it may modulate the presynaptic dopamine dysfunction observed in patients with schizophrenia [71].

The ability of acute ulotaront administration to regulate rat brain expression of the activity-regulated cytoskeleton-associated protein (*Arc*) and *c-Fos*, an immediate-early gene involved in neuroplasticity, memory formation, and sustaining cognitive processes, was tested. Following ulotaront administration, *Arc* and *c-Fos* mRNA levels were significantly upregulated in the prefrontal cortex and ventral hippocampus, but not in the striatum or dorsal hippocampus. At the same time, ulotaront attenuated increased *Arc* expression in the prefrontal cortex following acute PCP administration. Furthermore, mRNA levels of *Zif268/Egr1* (involved in several neuronal plasticity processes) and *Npas4* (a neuronal transcription factor that regulates the excitatory–inhibitory balance) were also significantly upregulated by acute ulotaront treatment in the PFC but not in other brain regions [61].

A summary of the experimental animal studies is presented in Table 1.

### 5.3. Pharmacokinetics and Metabolism

#### 5.3.1. Pharmacokinetics in Experimental Animals

The pharmacokinetics for intravenous (i/v) and per os (p/o) administration of ulotaront was preclinically assessed in male ICR mice (10 mg/kg, p/o), Sprague Dawley rats (5 and 10 mg/kg, p/o and i/v), and rhesus macaques (10 mg/kg, p/o and i/v) [17].

In mice, the C_max_ for 10 mg/kg, p/o, was 2854 ± 298 ng/mL and 7972 ± 2908 ng/g in plasma and the brain, respectively; the T_max_ was 30 min for plasma and 15 min for brain tissue; and the T_1/2_ was 0.847 h and 0.808 h in plasma and the brain, respectively.

In rats, following 10 mg/kg, p/o, the C_max_ was 1750 ± 369 ng/mL and 3762 ± 1324 ng/g in plasma and the brain, respectively; the T_max_ values for plasma and brain tissue were equal at 15 min; and the T_1/2_ was 2.1 h and 2.33 h in plasma and the brain, respectively. In rats, following 5 mg/kg, i/v, and 5 mg/kg, p/o, the C_max_ in plasma was 2578 ± 110 ng/mL and 1056 ± 173 ng/g; the T_max_ was 0.083 and 0.42 ± 0.14 h; and the T_1/2_ in plasma was 1.17 ± 0.16 h and 1.24 ± 0.1 h, respectively.

In monkeys, following 5 mg/kg, p/o, the C_max_ in plasma was 431 ± 104 ng/mL, the T_max_ was 6.00 ± 2.83 h, and the T_1/2_ was 3.03 h. In monkeys, following 5 mg/kg, i/v, the C_max_ in plasma was 2191 ± 194 ng/mL, the T_max_ was 0.083, and the T_1/2_ was 3.14 ± 1.26 h. The mean residence time was 5.90 h [17].

Thus, in experimental animals, ulotaront is rapidly absorbed, has a good bioavailability (~100% in rats, 92% in dogs, and 71% in monkeys), and tends to concentrate in brain tissue (brain concentration and brain AUC were approximately three times higher than in plasma). In follow-up in vitro ADME and preclinical pharmacokinetic studies, a high solubility and permeability for ulotaront has been also demonstrated [72]. In this study, ulotaront demonstrated low binding to animal and human plasma proteins, with an unbound fraction greater than 78% (in both animals and humans) [72]. Ulotaront exhibited low-to-moderate hepatic clearance in mouse, rat, monkey, and human hepatocytes [72]. Ulotaront’s hepatic clearance formation is mainly determined by CYP2D6, and both NADPH-dependent and NADPH-independent pathways seem to be involved in its metabolism [72]. The major metabolite identified in the plasma of mice, rats, rabbits, dogs, monkeys, and humans after a single dose or repeat dose of ulotaront is SEP-383103 [72].

#### 5.3.2. Pharmacokinetics in Humans

The population pharmacokinetics of ulotaront in adult subjects was analyzed using pooled data from seven phase 1 studies, one phase 2 acute study, and one 6-month extension study. Pharmacokinetic parameters were evaluated in men and women aged 18 to 55 years. Data were obtained from healthy volunteers (n = 99) and patients with schizophrenia (n = 305). A total of 53.7% of the tested subjects were White, 31.4% were Black, 10.9% were Asian, and 3.9% were other/mixed race. Over 80% of the Asian subjects in the analysis were Japanese [73]. Single and multiple oral doses (5–150 mg/day) were used. According to the pharmacokinetic analysis, ulotaront is well absorbed when taken orally. Ulotaront demonstrated dose proportionality for doses ranging from 25 to 100 mg, the mean maximum concentration, the area under the concentration–time curve, and the minimum concentration. The estimated median T_max_ was 2.8 h, and the median effective half-life was 7 h, leading to an accumulation ratio of 1.1 upon daily dosing. There were no major alterations in pharmacokinetic parameters after up to 12 weeks of daily dose administration. The pharmacokinetic parameters of Ulotaront were independent of sex, race, age, formulation, or the presence of schizophrenia. Only the body weight of the patients influenced ulotaront pharmacokinetics. Since CYP2D6 is involved in the metabolism of ulotaront, CYP2D6 metabolizer status could potentially affect pharmacokinetics, but due to the small sample size it is not yet possible to unequivocally answer this question. Taken together, these data indicate that ulotaront has a pharmacokinetic profile that is consistent with once-a-day dose administration [74]. The bioequivalence of tablet and capsule formulations of ulotaront was assessed, with no significant differences revealed [75]. Furthermore, no effect of food on the pharmacokinetics of the tablet form in humans was found [75].

### 5.4. Clinical Studies

Through the search of clinicaltrials.gov with the drug name “SEP-363856”, twenty-six trials were found. At the time of writing this article, twelve of these were completed, including one phase 3 study, and thirteen are ongoing, nine of which are phase 3 studies. Full information about the clinical trials of ulotaront is available in Table 2. Most studies focus on the effects of ulotaront in patients with schizophrenia. One study was conducted on patients with Parkinson’s disease psychosis, one on patients with major depressive disorder, one on patients with generalized anxiety disorder, and one on patients with narcolepsy. In the trial of ulotaront for narcolepsy, changes in the total number of cataplexy attacks over the 2-week treatment period were assessed. In most studies, Ulotaront is compared with a placebo, but in one phase 3 trial it will be compared with an extended-release form of quetiapine and in one phase 1 trial it was compared with amisulpride.

The results of the clinical trial NCT02969382, “A Study to Evaluate the Efficacy and Safety of SEP-363856 in Acutely Psychotic Adults with Schizophrenia”, and a long-term extension study are presented in two reports [53,73]. In the initial 4-week trial, the patients with an acute exacerbation of schizophrenia (120 patients were assigned to the ulotaront group and 125 to the placebo group) were randomly assigned to receive once-daily treatment with ulotaront (50 mg or 75 mg) or a placebo for 4 weeks. The mean total score on the PANSS changed by −17.2 points and −9.7 points in the ulotaront-treated and placebo-treated groups, respectively, indicating that ulotaront treatment resulted in a greater reduction in the PANSS total score from the baseline than the placebo. Importantly, besides the decrease in positive symptoms, a reduction in negative symptoms was demonstrated following ulotaront therapy. At the same time, adverse events with ulotaront were minimal and included somnolence and gastrointestinal symptoms. No difference between trial groups was noted for the incidence of extrapyramidal symptoms and the levels of lipids, glycated hemoglobin, and prolactin [73]. A follow-up 6-month extension study was performed to evaluate the safety and effectiveness of longer-term treatment with ulotaront. Patients with acute exacerbation of schizophrenia who completed a 4-week trial were given the option to enroll in an extension study involving open-label treatment with flexible doses (25/50/75 mg/d) of ulotaront for 6 months. The primary aim was to evaluate safety, while effectiveness outcomes were secondary and were determined as PANSS total scores and Brief Negative Symptom Scale (BNSS) total scores. A total of 156 patients were included in the open-label extension study and received at least one dose of ulotaront. After 26 weeks, the study completion rate was 66.9%; there were no deaths in the study. Six months of treatment with ulotaront resulted in continued improvement from baseline in the PANSS total (−22.6) and BNSS total (−11.3) scores, indicating the effectiveness of the drug with respect to both positive and negative symptoms. The most frequently reported adverse events were worsening of schizophrenia, headaches, insomnia, and anxiety. At the same time, ulotaront demonstrated minimal risk of extrapyramidal symptom development, with essentially no effect on body weight, lipids, glycemic indices, or prolactin. Also, ulotaront did not cause a prolongation of the corrected QT interval, calculated with the use of Fridericia’s formula (QTcF), or an increase in the QTcF interval of more than 60 msec [53]. Furthermore, improvement in overall functioning was detected using the University of California, San Diego, Performance-Based Skills Assessment (UPSA-B) scale, and small but consistent improvements in cognition were noted in the Cogstate composite and subscale task scores [76]. The results of the clinical trial NCT04369391, “A Clinical Study to Investigate the Effect of an Investigational Drug on Electrocardiogram Intervals in Adults with Schizophrenia”, were also reported [77]. In this randomized, single-dose, three-period crossover study (ulotaront 150 mg, placebo, moxifloxacin 400 mg), sixty subjects with schizophrenia were investigated. Ulotaront showed no clinically relevant effect on heart rate or electrocardiogram intervals, indicating that ulotaront is unlikely to cause clinically relevant cardiological side effects in patients with schizophrenia at a potentially maximum therapeutic dose [77]. Taken together, these data indicate that ulotaront has a unique risk–benefit profile compared to the current class of medications used to treat schizophrenia.

The results of the study on the efficacy and safety of ulotaront in patients with Parkinson’s disease psychosis (PDP) were presented at a conference [78] and recently published [79]. In this controlled study (ClinicalTrials.gov identifier: NCT02969369), 38 patients with PDP (ulotaront, n = 24; placebo, n = 14) were randomized to flexibly receive a dose of ulotaront (25, 50, or 75 mg/day) or placebo for 6 weeks. In this proof-of-concept study, improvements across several measures of the Scale for Assessment of Positive Symptoms—Parkinson’s Disease (SAPS-PD) were noted, without worsening of motor parkinsonism; however, due to the low number of subjects in the groups, the data do not have strong statistical power. Ulotaront was generally well tolerated. Further studies are warranted to validate ulotaront as a novel non-D2-receptor blocking treatment for PDP, particularly for patients with cognitive impairment [79].

Since, in rodents, ulotaront strongly suppressed rapid eye movement (REM) sleep [17], the ability of the drug to affect sleep architecture in healthy subjects was evaluated [80]. The effects of single oral doses (50 and 10 mg) of ulotaront on REM sleep were investigated in healthy male subjects (N = 12 for each dose) in a randomized, double-blinded, placebo-controlled, two-way crossover study. Plasma drug concentrations were also analyzed. Ulotaront suppressed REM sleep parameters with large effect sizes (>3) following single doses of 50 mg and plasma concentrations ≥100 ng/mL. Below that effective concentration, the 10 mg dose elicited much smaller effects, increasing only the latency to REM sleep, with an effect size of 1, and not time spent in REM. Ulotaront treatment at a dose of 50 mg was associated with a small increase in time spent in NREM (stage 2) and NREM (stage 3 (slow-wave)) sleep [80]. The results of post hoc exploratory analyses revealed a significant effect of ulotaront on quantitative REM sleep without atonia in healthy subjects following a 50 but not a 10 mg dose [81]. Recently, the results of a multicenter, double-blinded, placebo-controlled, randomized, phase 1b crossover trial (NCT05015673) comparing two doses of ulotaront with a placebo in subjects with narcolepsy–cataplexy were published [82]. Sixteen adults with narcolepsy–cataplexy received two daily oral doses of ulotaront (25 mg and 50 mg) for 2 weeks and were compared with a matching placebo group. Acute treatment with both doses of ulotaront significantly reduced the number of minutes spent in nighttime REM in patients, while sustained 2-week administration of both doses of ulotaront reduced the mean number of short-onset REM periods during the daytime multiple sleep latency test. At the same time, no significant improvement in patient or clinician measures of sleepiness was found in any treatment group, indicating no clinically meaningful effect in narcolepsy–cataplexy.

## 6. Conclusions

Based on a review of currently available preclinical and clinical studies, the main characteristics of ulotaront and its distinctive features were identified.

Ulotaront is a potent TAAR1 agonist with 5-HT1A receptor agonist activity that does not block D2/5-HT2a receptors. In preclinical studies, ulotaront demonstrated potential antipsychotic activity in several experimental animal models. In 4-week and 26-week studies, ulotaront was well tolerated following oral administration and showed an elimination half-life compatible with a once-daily regimen. In a phase 2 clinical trial, ulotaront showed efficacy against both positive and negative symptoms of schizophrenia, with minimal extrapyramidal and metabolic side effects. Ulotaront is the first investigational antipsychotic drug that does not act on D2 dopamine receptors but is effective in schizophrenia patients in clinical trials. Ulotaront is the first TAAR1 agonist for which phase 2 clinical trials have been completed and demonstrates efficacy in the treatment of patients with schizophrenia. If approved, ulotaront has the potential to be the first non-D2 medication for the treatment of schizophrenia. With increasing knowledge of the safety and efficacy of ulotaront in schizophrenia and the first clinical results on its potential efficacy in PDP, depression, and anxiety, further increase in its application to other neuropsychiatric disorders could be predicted.

## 7. Limitations and Future Directions

The major limitation of this work is that very limited information on ulotaront is currently available. The accumulation of knowledge of the mechanism of action and the efficacy of the drug through clinical studies currently being performed will hopefully result in the approval of ulotaront for clinical use in schizophrenia, and its application in a variety of other neuropsychiatric disorders might be expected.

## Figures and Tables

**Table 1:** Summary of experimental animal studies with ulotaront.

Method	Species	Ulotaront Doses (mg/kg)	Route of Administration	Effect	Reference
**Effects of in vivo methods related to antipsychotic actions and common adverse reactions**					
PCP-induced hyperlocomotion	C57BL/6J mice (♂) treated with PCP (5 mg/kg, i/p)	0, 0.3 *, 1 *, 3 *	p/o	↓	[17]
	C57BL/6J mice (♂) treated with PCP (5 mg/kg, i/p) and WAY-100635 (1 mg/kg, i/p)	2	p/o	0	
	Lister hooded rats (♀) treated with PCP (2.0 mg/kg, i/p)	0, 1 *, 3 *, 10 *	p/o	↓	[61]
MK-801-induced hyperlocomotion	WT mice treated with MK-801 (0.4 mg/kg, i/p)	10	p/o	↓	[56]
	TAAR1-KO mice treated with MK-801 (0.4 mg/kg, i/p)	10	p/o	↓	
	ICR mice treated with MK-801 (0.3 mg/kg, i/p)	0, 0.3, 1 *, 3 *	p/o	↓	[62]
	ICR mice treated with MK-801 (0.3 mg/kg, i/p) and olanzapine (0.1 mg/kg, p/o)	1	p/o	↓ (Booster effect)	
Amphetamine-induced hyperlocomotion	Lister hooded rats (♀) treated with d-amphetamine (0.1 mg/kg, i/p)	0, 1, 3, 10	p/o	0	[61]
	WT mice treated with d-amphetamine (5 mg/kg, i/p)	10	p/o	0	[61]
	TAAR1-KO mice treated with d-amphetamine (5 mg/kg, i/p)	10	p/o	0	
Cocaine (20 mg/kg)-induced hyperactivity	WT mice	10	p/o	0	[61]
	KO mice	10	p/o	0	
Apomorphine (1 mg/kg, s/c)-induced climbing	ICR mice	0, 1, 3, 10	p/o	0	[62]
	ICR mice treated with olanzapine (0.225, 0.45, 0.9 mg/kg, p/o)	2.275, 4.55 *, 9.1 *	p/o	↓ (Booster effect)	
Acoustic startle response	C57BL/6J mice (♂)	0, 0.3, 1, 3, 10, 30	p/o	0	[17]
Prepulse inhibition test	C57BL/6J mice (♂)	0, 0.3, 1, 3 *, 10 *, 30 *	p/o	↑	[17]
	WT mice	10	p/o	↑	[56]
	TAAR1-KO mice	10	p/o	0	
MK-801 (0.4 mg/kg, i/p)-induced disruption of prepulse inhibition	WT mice	10	p/o	↑	[56]
	TAAR1-KO mice	10	p/o	0	
PCP (5 days, 2 mg/kg, s/c)-induced deficits in social interaction	Sprague Dawley rats (♂)	0, 1 *, 3 *, 10 *	p/o	↓	[17]
Cognitive deficits induced by sub-chronic PCP (7 days, 2 mg/kg, i/p) administration	Lister hooded rats (♀)	0, 1, 10 *	p/o	↓	[61]
MK-801 (6-days, 1 mg/kg)-induced increase in escape latency in Morris water maze	ICR mice	0, 0.3 *, 1 *, 3 *	p/o	↓	[62]
	ICR mice treated with olanzapine (0.05 mg/kg, p/o)	0.3	p/o	↓ (Booster effect)	
Locomotor activity in the home cage	Sprague Dawley rats (♂)	0, 1, 3, 10	p/o	0	[17]
Locomotor activity in open field	C57BL/6J mice (♂)	0, 0.3, 1, 3 *	p/o	↓	[17]
	Sprague Dawley rats (♂)	0, 1 *, 3 *, 10 *	p/o	↓	[61]
	WT mice	10	p/o	↓	[56]
	KO	10	p/o	0	
Bar test (catalepsy)	C57BL/6J mice (♂)	0, 100	p/o	0	[17]
Weight	ICR mice	2, 3, for 34 days	p/o	0	[62]
Olanzapine (34 days, 3 mg/kg, p/o)-induced weight gain		2	p/o	↓	
**Effects of in vivo methods related to other neuropsychiatric disorders**					
Forced swim test (immobility time)	C57BL/6J mice (♂)	0, 0.3, 1 *, 3 *, 10 *	p/o	↓	[17]
	ICR mice (♂)	0, 0.1, 0.3 *, 1 *, 3, 10 *	p/o	↓	[68]
	ICR mice (♂) treated with duloxetine (3, 5, 7.5, 10 mg/kg, p/o)	0.1 *	p/o	↓ (Booster effect)	
Tail suspension test (immobility time)	ICR mice (♂)	0, 0.1, 0.3 *, 1, 3, 10	p/o	↓	[68]
	ICR mice (♂) treated with duloxetine (3, 5, 7.5, 10 mg/kg, p/o)	0.1 *	p/o	↓ (Booster effect)	
Deficits in sucrose preference test inducedby the 14-day chronic unpredictable mild stress procedure	ICR mice (♂)	15	p/o	↓	[68]
	ICR mice (♂) treated with duloxetine (15 mg/kg, p/o)	15	p/o	↓ (Booster effect)	
Food-reinforced lever pressing (fixed ratio 10; response rate)	Sprague Dawley rats (♂)	0, 3 *, 10 *	i/v	↓	[69]
Food-reinforced lever pressing (fixed ratio 3; response rate)	Sprague Dawley rats (♂)	0, 0.3, 1, 3 *	i/v	↓	[69]
Substitution test in cocaine self-administration	Sprague Dawley rats (♂)	0, 0.3, 1, 3	i/v	0	[69]
Substitution test in amphetamine self-administration	Sprague Dawley rats (♂)	0, 0.3, 1, 3	i/v	0	[69]
Substitution test in heroin self-administration	Sprague Dawley rats (♂)	0, 0.3, 1, 3	i/v	0	[69]
Generalization in amphetamine drug discrimination	Sprague Dawley rats (♂)	0, 3, 10, 30	p/o	0	[69]
Generalization in MDMA drug discrimination	Lister hooded rats (♀)	0, 3, 10, 30 *	p/o	Substitutes	[69]
Prime-induced cocaine self-administration	Long–Evans rats (♂)	0, 1, 3, 10	p/o	0	[69]
Que-induced cocaine self-administration	Long–Evans rats (♂)	0, 1, 3, 10 *	p/o	↓	[69]
Core body temperature	WT mice	10	p/o	↓	[56]
	TAAR1-KO mice			↓ (Effect is lower than in WTs)	
REM sleep (duration, latency)	Sprague Dawley rats (♂)	0, 1 *, 3 *, 10 *	p/o	↓	[17]
Non-REM sleep		0, 1, 3, 10	p/o	0	

0—no effects; ↓—decrease; ↑—increase; *—significant vs. control according to post hoc comparisons.

**Table 2:** Clinical trials evaluating Ulotaront (SEP-363856).

Title	NCT Number	Status	Conditions	Interventions	Phases	Enrollment	Start Date
**Schizophrenia**							
A Study Assessing the Safety, Tolerability, and Pharmacokinetics of SEP-363856 in Male and Female Subjects with Schizophrenia	NCT01940159	Completed	Schizophrenia	SEP-363856 vs. placebo	Phase 1	48	1 August 2013
Study Assessing the Safety, Tolerability, and Pharmacokinetics of SEP-363856 in Male and Female Subjects with Schizophrenia	NCT01994473	Completed	Schizophrenia	SEP-363856 vs. placebo	Phase 1	48	1 October 2013
Study Assessing SEP-363856 in Male and Female Volunteers with High or Low Schizotype Characteristics	NCT01972711	Completed	Schizophrenia	SEP-363856 50 mg vs. Amisulpride 400 mg vs. placebo	Phase 1	105	1 March 2014
A Study to Evaluate the Efficacy and Safety of SEP-363856 in Acutely Psychotic Adults with Schizophrenia	NCT02969382	Completed	Schizophrenia	SEP-363856 vs. placebo	Phase 2	245	5 December 2016
An Extension Study of Safety and Tolerability of SEP-363856 in Adult Subjects with Schizophrenia	NCT02970929	Completed	Schizophrenia	SEP-363856	Phase 2	157	31 January 2017
Study Assessing the Safety, Tolerability, and Pharmacokinetics of SEP-363856 in Japanese Male and Female Subjects with Schizophrenia in 2 Parts	NCT03370640	Completed	Schizophrenia	SEP-363856	Phase 1	24	29 November 2017
A Study of the Long-term Safety and Tolerability of an Investigational Drug in People with Schizophrenia	NCT04115319	Completed	Schizophrenia	SEP-363856 vs. quetiapine XR	Phase 3	300	15 November 2019
A Clinical Study to Investigate the Effect of an Investigational Drug on Electrocardiogram Intervals in Adults with Schizophrenia	NCT04369391	Completed	Schizophrenia	SEP-363856 150 mg vs. placebo vs. Moxifloxacin 400 mg	Phase 1	150	18 June 2020
Study Assessing the Safety, Tolerability, and Pharmacokinetics of SEP-363856 in Japanese Male and Female Subjects with Schizophrenia	NCT04325737	Completed	Schizophrenia	SEP-363856 vs. placebo	Phase 1	32	31 March 2020
A Clinical Trial Study to Determine the Effect of an Investigational Drug (SEP-363856) Has on the Way That the Drug Metformin Travels Through the Body in People with Schizophrenia	NCT04865835	Completed	Schizophrenia	Metformin 850 mg + placebo vs. Metformin 850 mg + SEP-363856	Phase 1	24	12 May 2021
A Clinical Study to Investigate the Effect of an Investigational Drug as an Added Medication to an Antipsychotic, in Adults With Schizophrenia, as Measured Positron Emission Tomography (PET) Imaging	NCT04038957	Recruiting	Schizophrenia	SEP-363856	Phase 1	22	7 August 2019
A Clinical Trial to Study the Efficacy and Safety of an Investigational Drug in Acutely Psychotic People with Schizophrenia	NCT04072354	Recruiting	Schizophrenia	SEP-363856 50 mg vs. SEP-363856 75 mg vs. placebo	Phase 3	525	11 September 2019
A Clinical Trial That Will Study the Efficacy and Safety of an Investigational Drug in Acutely Psychotic People with Schizophrenia	NCT04092686	Recruiting	Schizophrenia	SEP-363856 75 mg vs. SEP-363856 100 mg vs. placebo	Phase 3	462	30 September 2019
A Clinical Study to Evaluate the Long-term Safety and Tolerability of an Investigational Drug in People with Schizophrenia	NCT04109950	Recruiting	Schizophrenia	SEP-363856	Phase 3	555	4 October 2019
A Clinical Trial to Evaluate the Efficacy and Safety of SEP-363856 in Acutely Psychotic People With Schizophrenia, Followed by an Open-label Extension Phase	NCT04825860	Recruiting	Schizophrenia	SEP-363856 50 mg vs. SEP-363856 75 mg vs. placebo	Phase 2/3	480	29 March 2021
A Clinical Trial to Evaluate the Long-term Safety and Tolerability of SEP-363856 in Patients With Schizophrenia in Japan	NCT05359081	Recruiting	Schizophrenia	Flexible dose of SEP-363856 (50 mg/day and 75 mg/day)	Phase 3	220	16 April 2022
A Clinical Study That Will Assess How Food Moves Through the Stomach and Effects Blood Glucose Levels in Subjects With Schizophrenia Taking SEP-363856 or and Prior Antipsychotic (PA) Standard	NCT05402111	Recruiting	Schizophrenia	SEP-363856 (25 and 50 mg) vs. risperidone, olanzapine, quetiapine, or aripiprazole	Phase 1	36	13 June 2022
A Clinical Study That Will Assess the Effect of SEP-363856 and Prior Antipsychotic (PA) Standard of Care on Glucose and Regulation of Insulin in Patients With Schizophrenia	NCT05463770	Recruiting	Schizophrenia	SEP-363856, 12.5 mg, 25 mg, and 50 mg tablets vs. Prior Antipsychotic (PA) Standard of Care in Subjects with Schizophrenia Suffering from Metabolic Dysregulation	Phase 1	24	30 August 2022
A Clinical Study That Will Assess the Effect of SEP-363856 or Prior Antipsychotic (PA) Standard of Care on Body-weight Associated Parameters in Subjects With Schizophrenia	NCT05542264	Recruiting	Schizophrenia	SEP-363856 flexible dose vs. risperidone, olanzapine, quetiapine, or aripiprazole	Phase 1	60	15 November 2022
A Clinical Study That Will Evaluate How Well SEP-363856 Works and How Safe it is in People With Schizophrenia That Switch to SEP-363856 From Their Current Antipsychotic Medication	NCT05628103	Recruiting	Schizophrenia	SEP-363856 flexibly dosed	Phase 3	120	19 December 2022
An Extension Study to a Clinical Study That Will Continue to Evaluate the Effectiveness and Safety of SEP-363856 in People With Schizophrenia That Switch to SEP-363856 From Their From Their Current Antipsychotic Medication	NCT05741528	Not yet recruiting	Schizophrenia	SEP-363856 tablet	Phase 3	67	20 February 2023
A Clinical Study to Learn if SEP-363856 Has Physical Dependence in Adults With Schizophrenia	NCT05848700	Not yet recruiting	Schizophrenia	SEP-363856 tablet vs. placebo	Phase 3	60	8 May 2023
**Other neuropsychiatric disorders, including non-schizophrenic psychoses**							
A Study to Evaluate the Efficacy, Safety and Tolerability of SEP-363856 in Subjects with Parkinson’s Disease Psychosis	NCT02969369	Completed	Parkinson’s disease psychosis	SEP-363856 vs. placebo	Phase 2	39	31 December 2016
This is a Study to Determine the Effect of Multiple Doses of an Investigational Drug, Taken by Mouth, in People With Narcolepsy-cataplexy	NCT05015673	Completed	Narcolepsy	SEP363856 vs. placebo	Phase 1	18	5 June 2014
A Trial of the Safety and Efficacy of SEP-363856 in the Treatment of Adults With Major Depressive Disorder	NCT05593029	Recruiting	Major depressive disorder	SEP-363856 + antidepressant therapy vs. placebo + antidepressant therapy	Phase 2/3	900	9 November 2022
A Clinical Study That Will Meaure How Well SEP-363856 Works and How Safe it is in Adults With Generalized Anxiety Disorder	NCT05729373	Recruiting	Generalized anxiety disorder	SEP-363856 (50–75 mg/day) vs. placebo	Phase 2/3	434	8 March 2023

## Data Availability

Not applicable.
